# The heuristics-and-biases inventory: An open-source tool to explore individual differences in rationality

**DOI:** 10.3389/fpsyg.2023.1145246

**Published:** 2023-04-03

**Authors:** Vincent Berthet, Vincent de Gardelle

**Affiliations:** ^1^Department of Psychology, Université de Lorraine, 2LPN, Nancy, France; ^2^Centre d’Économie de la Sorbonne, CNRS UMR 8174, Paris, France; ^3^CNRS and Paris School of Economics, Paris, France

**Keywords:** individual differences, heuristics and biases, rationality, decision-making, measures

## Abstract

Over the last two decades, there has been a growing interest in the study of individual differences in how people’s judgments and decisions deviate from normative standards. We conducted a systematic review of heuristics-and-biases tasks for which individual differences and their reliability were measured, which resulted in 41 biases measured over 108 studies, and suggested that reliable measures are still needed for some biases described in the literature. To encourage and facilitate future studies on heuristics and biases, we centralized the task materials in an online resource: The Heuristics-and-Biases Inventory (HBI; https://sites.google.com/view/hbiproject). We discuss how this inventory might help research progress on major issues such as the structure of rationality (single vs. multiple factors) and how biases relate to cognitive ability, personality, and real-world outcomes. We also consider how future research should improve and expand the HBI.

## 1. Introduction

The heuristics-and-biases (HB) research program, introduced by Tversky and Kahneman in the early 1970s (Kahneman and Tversky, 1972; Tversky and Kahneman, 1973, 1974), is a descriptive approach to decision-making that consists of invoking heuristics (mental shortcuts) to explain systematic deviations from rational choice behavior. For instance, people may misestimate a numerical value because of an overreliance on information that comes to mind and insufficient adjustment (anchoring-and-adjustment heuristic; Tversky and Kahneman, 1974). Another well-known example of a cognitive bias is the framing effect, by which individuals respond differently to a choice problem when the possible outcomes are framed as gains or as losses.

Since its inception, research on HB has produced a large literature on errors in judgment and decision-making (Gilovich et al., 2002) and triggered much discussion. Important questions include, among others, whether deviations from rationality can be reduced to randomness in choice (Stanovich and West, 2000), and whether HB effects are universal or instead vary across situations (e.g., due to the ecological or non-ecological nature of the task; Gigerenzer, 1996, 2008) or across individuals (Stanovich and West, 1998; Baron, 2008). Indeed, not all HB effects are present to the same extent in all individuals. Some biases are more prevalent than others: loss aversion might be found in a large majority of individuals (Gächter et al., 2022), whereas framing effects might not (Li and Liu, 2008). Some individuals might be more susceptible than others. In the case of attribution bias, for instance, that is the observation that individuals are more prone to credit themselves for positive than for negative events, a large meta-analysis conducted by Mezulis et al. (2004) demonstrated significant variations across countries, across genders, as well as associations with clinical symptoms.

In addition, to go beyond establishing a list of biases, efforts have been made to describe how different biases relate to each other. In this line of work, some studies have argued for a common decision-making competence underlying several HB tasks (Bruine de Bruin et al., 2007), akin to the *g*-factor (Carroll, 1993), whereas other studies covering a more heterogeneous set of tasks have provided support for a more complex, multidimensional structure (Klaczynski, 2001; Weaver and Stewart, 2012; Aczel et al., 2015; Teovanović et al., 2015; Ceschi et al., 2019; Berthet et al., 2022; Erceg et al., 2022; Rieger et al., 2022; Burgoyne et al., 2023). This research illustrates how the cognitive structure underlying heuristics and biases in decision-making can be investigated using individual differences.

Individual differences, however, have not been the main focus of earlier research on HB effects. The first reason is that the goal of this research was to demonstrate the existence of HB effects in the first place, on average, across participants. A second reason was the methodological choice to do so using between-subjects designs. This choice was notably motivated by the assumption that between-subjects designs favor spontaneous, intuitive answers in individuals, which are precisely the phenomenon of interest in HB research. As Kahneman (2000, p. 682) puts it: “much of life resembles a between-subjects experiment.” By contrast, within-subject designs would be more transparent to participants, emphasizing the comparison between the conditions of interest, which might trigger the engagement of a slower, more deliberative system, and the override of intuitive answers, thereby reducing HB effects (Kahneman and Tversky, 2000; Kahneman and Frederick, 2005).

Critically though, the assumption that within-subject designs would produce smaller HB effects has not always been supported in empirical studies (Piñon and Gambara, 2005; Aczel et al., 2018; Gächter et al., 2022). In addition, regarding transparency, participants may remain unable to identify the research hypothesis in within-subject designs (Lambdin and Shaffer, 2009). In addition, they offer better statistical power than between-subject designs, and they eliminate confounds related to potential differences between participants in the different experimental conditions. Thus, within-subject designs seem appropriate tools to examine HB effects. As they allow for measuring biases at the individual level, these designs are particularly suited for individual differences. However, the measurement of individual differences in HB raises a practical and methodological issue: Finding such measures can be difficult and time-consuming while we still do not know much about their reliability.

The goal of the present study is to address these issues. First, we identify the currently available tasks that measure HB effects at the individual level. To do so, we conduct a systematic survey of empirical studies measuring one or more cognitive biases in a within-subject manner, focusing on studies in which the reliability of the measure used to quantify the bias is documented. Indeed, tasks optimized for large average effects might turn out to be less reliable at the individual level, producing a tradeoff between effect size and reliability (Hedge et al., 2018). We find that when reliability is documented, it is usually good. However, there are also a good number of HB effects for which no within-subject design has been tested or for which reliability is not known.

Second, we introduce an open online resource for the scientific community: the Heuristics-and-Biases Inventory (HBI^1^). This platform aims primarily at providing in a single location the experimental material for quantifying HB effects at the single subject level. The platform is meant to include new tasks as they are developed. Our hope is that this contribution will foster research on individual differences regarding cognitive heuristics and biases.

## 2. Methods

We conducted a systematic review in accordance with the PRISMA guidelines (Page et al., 2021).^2^

### 2.1. Search strategy

The following databases were searched for peer-reviewed empirical articles in June 2022: Web of Science, PsycINFO, and Pubmed. Our search strategy was based on the conjunction of two criteria: (1) the presence of “individual differences” in the title or in the abstract and (2) the presence of the terms “heuristics and biases” OR “cognitive bias” OR “cognitive biases” OR “behavioral biases” OR “rationality” OR “Decision-Making Competence” in the title or the abstract. All entries were imported in Zotero to remove duplicates, after which titles and abstracts were screened independently by two coders, according to predefined eligibility criteria.

Noteworthy, this search strategy had two implications. First, we likely missed relevant papers as we did not enter every single HB as a keyword, thereby limiting the comprehensiveness of our inventory. Second, our search strategy would not necessarily filter out studies that addressed psychological biases other than those pertaining to the heuristics-and-biases tradition (judgment and decision-making) such as health anxiety-related biases (e.g., interpretive bias and negativity bias) and the cognitive bias modification paradigm which aims at reducing them (e.g., Hallion and Ruscio, 2011).

### 2.2. Inclusion and exclusion criteria for studies

Included studies had to (1) be published in peer-reviewed scientific journals, (2) be written in English, and (3) be conducted on human participants. Reviews, conceptual or theoretical articles, book chapters, conference proceedings, dissertations, and editorial materials were excluded. We also excluded as follows: (1) Studies that addressed biases not pertaining to the HB tradition (e.g., health anxiety-related biases and implicit biases) for reasons previously mentioned, (2) studies in which self-report (questionnaires) rather than behavioral measures were used, (3) studies that merely applied the Adult Decision-Making Competence (ADMC), (4) studies in which a between-subject design was used. In addition, we chose not to include in the inventory two biases related to risk aversion (ambiguity aversion and zero-risk bias), which refers to a preference rather than a rationality failure (refer to the Discussion section).

### 2.3. Data collection and analysis

Relevant data was extracted by VB. The following information was collected for each study: author names, year of publication, title and journal where the study was published, study design, number of participants, inclusion/exclusion criteria, the HB task(s) used, whether the task(s) included single or multiple items, and the estimated reliability when reported. Discrepancies that emerged after full-text screening were resolved through a consensus meeting.

## 3. Results

**Figure 1:**
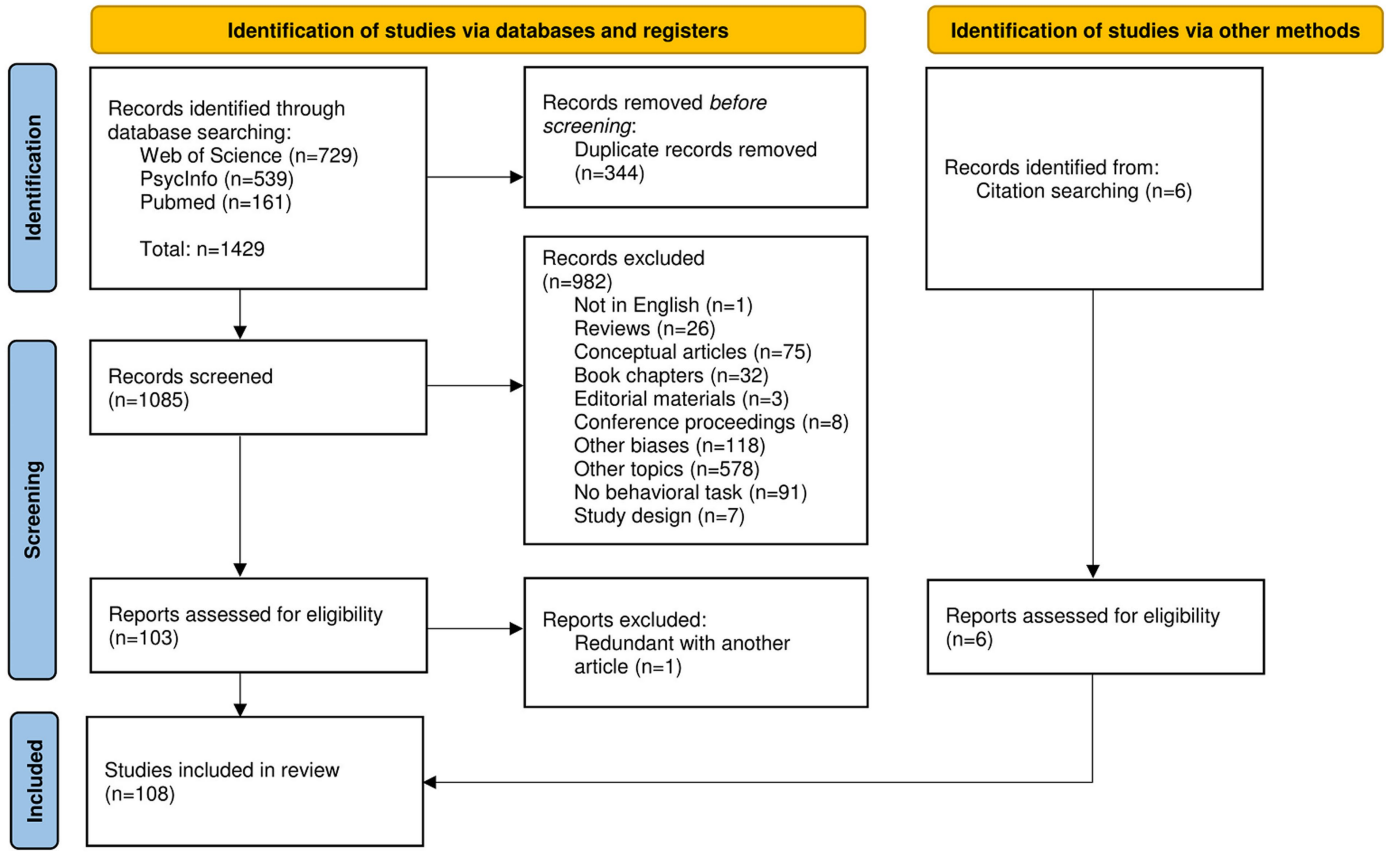
PRISMA flowchart of study selection.

Figure 1 displays the PRISMA flowchart with full detail of this process. The complete search resulted in 1429 articles, leaving 1,091 articles once duplicates were removed. After title and abstract screening, 109 articles met the inclusion criteria and were eligible for full-text assessment. One study was subsequently excluded because the author published the same data in another article. A total of 108 studies met eligibility criteria and were included in the review.

### 3.1. Study characteristics

Overall, the 108 studies included a total of 58,808 participants. Slightly more than half of the studies investigated a single HB (*n* = 56), while the rest addressed multiple HB (*n* = 51). Regarding the number of items, studies used one or several single-item tasks (*n* = 29), one or several multi-item tasks (*n* = 64), or both single and multi-item tasks (*n* = 14). Critically, out of the 78 studies that used multi-item tasks in the present survey, only 14 reported estimates of score reliability.

### 3.2. An inventory of tasks measuring heuristics and biases

Table 1 provides a reduced presentation of the outcome of this systematic survey. We identified 41 heuristics and biases for which there are tasks to measure individual differences. For each bias, we indicate the original paper introducing a typical task to measure the bias, the description of the task, the number of items, and estimated reliability when reported. A full version of the table is available in the supplementary material and on the HBI website, which also indicates, for each bias, the scoring rule and references of studies that merely used the task but did not report reliability. Note that there can be different measures available for some biases (e.g., anchoring) and that the same task can be associated with different scoring procedures (e.g., framing). The items for each task are available on the HBI website.

**Table 1:** Inventory and reliability of tasks measuring individual differences in heuristics and biases.

Task	Source	Items	Reliability
Anchoring heuristic: Tendency to adjust judgments toward the first piece of information.	Teovanović et al. (2015)	24	0.77
	Berthet (2021)	12	0.68
	Stanovich et al. (2016)	8	0.48
	Berthet et al. (2022)	8 pairs	0.67, 0.75, *r*_tt_ = 0.63
Attribution bias (including self-attribution bias): Tendency to refer to internal rather than external factors when explaining a person’s behavior.	None		
Availability heuristic: Tendency to judge events’ likelihood or frequency based on ease of recall.	Berthet et al. (2022)	4 pairs	0.67, 0.81, *r*_tt_ = 0.48
	Erceg et al. (2022)	4	0.77
Base-rate neglect (statistical): Tendency to ignore base rates in favor of individuating information.	Burič and Šrol (2020)	8	0.77, 0.78
	Šrol and De Neys (2021)	8	0.82
	Burgoyne et al. (2023)	11	0.46
	Šrol (2022)	4	0.70
	Erceg et al. (2022)	4	0.93, 0.95
	Berthet (2021)	4	0.70
Base-rate neglect (causal): Tendency to ignore causally relevant base rates in favor of individuating information.	Teovanović et al. (2015)	10	0.71
	Erceg et al. (2022)	3	0.55, 0.48
Belief bias in syllogistic reasoning: Tendency to evaluate deductive arguments based on the believability of the conclusion rather than its logical validity.	Teovanović et al. (2015)	8	0.76
	Berthet (2021)	4	−0.15
	Stanovich et al. (2016)	16	0.65
	Erceg et al. (2022)	8	0.79, 0.82
	Burič and Šrol (2020)	8	0.67, 0.78
	Šrol and De Neys (2021)	8	0.80
Better-than-average effect: Tendency to perceive one’s abilities as superior to the average.	Rieger et al. (2022)	3	0.60
Bias blind spot: Tendency to see themselves as less biased than other people.	Scopelliti et al. (2015)	14	0.86
Confirmation bias (four-card selection task): Tendency to confirm rather than infirm the hypothesis (logical rule) at hand.	Burgoyne et al. (2023)	10	0.66
	Erceg et al. (2022)	4	0.86, 0.80
	Berthet et al. (2022)	4	0.84
Confirmation bias (2–4-6 task): Tendency to confirm rather than infirm the hypothesis (numerical rule) at hand.	Berthet et al. (2022)	3	0.75
Confirmation bias (interviewee’s personality task): Tendency to confirm rather than infirm the hypothesis (personality trait) at hand.	Berthet (2021)	4	0.68
	Berthet et al. (2022)	4	0.83, 0.88, r_tt_ = 0.75
	Berthet et al. (2022)	4	0.64
Confirmation bias (financial decision-making) Tendency for people to disregard the counterevidence regarding their financial investments.	Rieger et al. (2022)	5	0.66
Conjunction fallacy: Tendency to judge that a conjunction of two possible events is more likely than one or both of the conjuncts.	Burgoyne et al. (2023)	7	0.69
	Šrol and De Neys (2021)	8	0.78
	Šrol (2022)	4	0.63
Conservatism: Tendency to overweight prior experience relative to new information.	None		
Covariation detection: Tendency for people to ignore essential comparative (control group) information.	None		
Debt account aversion: Tendency for consumers saddled with multiple debts to be motivated to reduce their total number of outstanding loans, rather than their total debt across loans.	None		
Denominator neglect/ratio bias: Tendency to pay too much attention to numerators and inadequate attention to denominators.	Stanovich et al. (2016)	12	0.88
Framing (risk and attribute): Tendency to be affected by how information is structured.	Bruine de Bruin et al. (2007)	14 pairs	0.62, r_tt_ = 0.58
	Parker and Fischhoff (2005)	5 pairs	0.30
	Stanovich et al. (2016)	11 pairs	0.66
	Berthet (2021)	8 pairs	0.74
	Berthet et al. (2022)	8 pairs	0.76, 0.85, *r*_tt_ = 0.45
	Erceg et al. (2022)	8 pairs	0.35, 0.17, 0.24
Fungibility of money: Tendency for people to ignore the fact that all money is the same.	None		
Gambler’s fallacy: Tendency to believe that the probability for an outcome after a series of outcomes is not the same as the probability for a single outcome.	Erceg et al. (2022)	4	0.76
	Šrol (2022)	4	0.52
Hindsight bias: Tendency to make different judgments (e.g., judging the probability of an outcome) between hindsight and foresight conditions.	Teovanović et al. (2015)	14	0.66
	Berthet (2021)	10	0.62
House money effect: Tendency for people to make decisions dependent on the prior gain or loss; includes greater tendency to gamble with recently won money.	None		
Illusion of Control: Tendency to overestimate their ability to control events.	None		
Insensitivity to sample size: Tendency to neglect sample size in inferential judgments.	None		
Irrational diversification: Tendency for people to favor a portfolio based on the perceived risk rather than the actual risk of the portfolio (based on real variance or probability).	None		
Loss Aversion: Tendency to prefer avoiding losses to acquiring equivalent gains.	None		
Mental accounting: Tendency to assign different mental values to the same sum of money.	None		
Money illusion: Tendency for people to think of money in nominal, rather than real, terms.	None		
Myside bias: Tendency to evaluate evidence, generate evidence, and test hypotheses in a manner biased toward their own prior opinions and attitudes.	None		
Omission bias: Tendency to avoid actions that carry some risk but prevent a larger risk.	None		
Outcome bias: Tendency to evaluate the quality of a decision based on its outcome.	Teovanović et al. (2015)	10 pairs	0.83
	Berthet (2021)	16	0.85^b^
	Berthet et al. (2022)	16	0.89^b^, 0.91^b^, *r*_tt_ = 0.78
	Erceg et al. (2022)	4 pairs	0.65, 0.68
Overconfidence: Tendency to overestimate their abilities.	Bruine de Bruin et al. (2007)	34	0.77, *r*_tt_ = 0.47
	Parker and Fischhoff (2005)	42	0.79
	Teovanović et al. (2015)	21	0.94
	Stanovich et al. (2016)	36	0.55
	Berthet (2021)	11	0.81^b^
	Berthet et al. (2022)	11	0.73^b^, 0.59^b^, *r*_tt_ = 0.54
	Hansson et al. (2008)	40, 40	0.84, 80
	Glaser et al. (2013)	15	0.83
Probability matching: Tendency to match choice proportions to outcome proportions in a binary prediction task.	Fletcher et al. (2011)	2	0.68
Probability neglect bias: Tendency for people to disregard the small probability of an outcome when facing a situation that arouses strong emotions.	None		
Proportion dominance: Preference for proportionally higher gains, such that the same absolute quantity is valued more as the reference group decreases (e.g., saving 10/10 lives is preferred to saving 10/100 lives).	None		
Regression to the mean: Tendency to neglect that extremely high or extremely low observations tend to become more moderate (i.e., closer to the mean) over time.	None		
Regret aversion: Tendency to make decisions in order to avoid feeling regret in future.	Rieger et al. (2022)	3	0.60
Representativeness heuristic: Tendency to assess similarity of objects and organize them based around the category prototype.	Yoon et al. (2021)	26, 26, 26	0.90, 0.86, 0.88
	Morsanyi et al. (2009)	3	0.51
Status quo bias (or default bias): Tendency to choose the default option.	None		
Sunk cost fallacy: Tendency to continue an endeavor once an investment in money, effort, or time has been made.	Bruine de Bruin et al. (2007)	10	0.54, *r*_tt_ = 0.61
	Parker and Fischhoff (2005)	2	0.03
	Berthet (2021)	5	0.35
	Berthet et al. (2022)	10	0.38
	Teovanović et al. (2015)	8	0.76
	Erceg et al. (2022)	4	0.56, 0.39
Temporal discounting: Tendency to prefer smaller immediate over larger delayed reward.	Stanovich et al. (2016)	26	0.97

Reliability is measured by Cronbach’s alpha, Spearman-Brown corrected split-half reliability (^b^), or test–retest correlation (*r*_tt_). Some studies reported several estimates of score reliability, which are all included in the table. “None” means that all studies that aimed to measure the bias used single-item tasks or that multi-item tasks were used, but the authors reported no estimates of reliability. Refer to the HBI website (https://sites.google.com/view/hbiproject/), for the full table.

It turns out that the list includes the main biases studied in HB research. In fact, 18 out of the 41 HB are among the biases that violate normative models listed by Baron (2008). Our review points out, however, that there has been no attempt to measure individual differences for several significant biases, such as planning fallacy and prominence effect.

### 3.3. Reliability

Reliability (internal consistency) can be only estimated when multi-item tasks are used. Although this was the case for 23 of the HB tasks identified here, only 14 of the reviewed studies reported estimates of internal consistency, and two studies assessed test–retest reliability (Bruine de Bruin et al., 2007; Berthet et al., 2022). For instance, for the status quo bias, or for the insensitivity to sample size, the reliabilities of the measures are unknown. In addition, 11 HB have been measured only with single-item tasks so far (ambiguity aversion, attribution bias, conservatism, denominator neglect, illusion of control, loss aversion, mental accounting, myside bias, omission bias, proportion dominance, and regression to the mean).

Based on the available estimates of internal consistency (excluding estimates of test–retest reliability which are too infrequent), the reliability of HB scores is most often above the generally accepted standard of 0.70 (Nunnally and Bernstein, 1994). This finding is noteworthy and confirms that despite the “reliability paradox” described by Hedge et al. (2018), tasks that were primarily designed to produce robust between-subject experimental effects can be turned into reliable measures of individual differences (note, however, that our estimate might be inflated by publication bias). Some exceptions are the framing effects and sunk cost fallacy, for which low reliabilities have been repeatedly found.

## 4. Discussion

The aim of the present study was to provide a systematic review of individual difference measures used in heuristics-and-biases research. Based on 108 studies, we listed 41 biases for which at least one behavioral task allows one to calculate individual scores. While it is apparent that some of the tasks belong to a particular category (e.g., availability heuristic, conjunction fallacy, gambler’s fallacy, probability matching, and base-rate neglect all assess biases in probability), we did not organize the tasks according to a particular theoretical taxonomy (e.g., Baron, 2008; Stanovich et al., 2008). Indeed, a key aim of the HBI is to help researchers build a robust empirical classification of HB by allowing them to include a large number of tasks in the study design and, therefore, to test the validity of the existing theoretical taxonomies (Refer to the following text).

Noteworthy, our review raised the issue of the reliability of such scores. Indeed, a significant number of HB have been measured only with single-item tasks, which does not allow checking reliability. When multi-item tasks are used, the reliability of scores is not systematically reported. In addition, low-reliability estimates have been repeatedly found for some biases (e.g., framing and sunk cost fallacy). However, based on the available estimates of internal consistency, the reliability of HB scores turns out to be most often above the generally accepted standard of 0.70. We encourage researchers to (1) use multi-item tasks and systematically report score reliability, (2) avoid calculating composite scores derived from single-item HB tasks as such scores are unreliable (West et al., 2008; Toplak et al., 2011; Aczel et al., 2015).

In the following subsections, we discuss the limits of our systematic review, how the HBI relates to existing taxonomies, and how it could be used to further address the impact of cognitive biases on real-life behavior.

### 4.1. Limitations of the systematic review

There are several limitations of this systematic review worth considering. First, the comprehensiveness of our inventory is limited by our search strategy. In order to cover all papers that addressed individual differences in HB, one should enter every single heuristic and bias as a keyword, which we did not do for practical reasons. Note, however, that our review was not meant to be exhaustive but rather to lay the foundation for listing HB tasks that are suited for the measurement of individual differences. As a collaborative and evolutive repository, the HBI may become more exhaustive over time.

The second limit relates to the selection of biases. As mentioned in the Methods section, we excluded psychological biases that do not fall within the category of heuristics and biases, defined as rationality failures. In particular, health anxiety-related biases such as interpretive bias (the tendency to inappropriately analyze ambiguous stimuli) and negativity bias (the tendency to pay more attention or give more weight to negative experiences over neutral or positive experiences) are typically not considered in the classification of biases in the heuristics-and-biases approach (Baron, 2008). Similarly, we did not include in our inventory two biases related to risk aversion (ambiguity aversion and zero-risk bias), which refers to a preference rather than a rationality failure (an individual is considered risk averse if she prefers a certain or risky option to a riskier option with equal or higher expected value while an individual who prefers a risky option to a certain or less risky option with higher expected value will be considered risk-seeking; Fox et al., 2015). However, one could argue that the exclusion of such biases is somewhat arbitrary as there is no objective criterion to qualify a bias under the heuristics-and-biases approach. Based on how the HBI is used by researchers, we will consider the possibility of expanding the scope of the inventory to include other types of biases.

### 4.2. HBI and existing inventories

We discuss here how the HBI compares with two related tools, the ADMC and the Comprehensive Assessment of Rational Thinking (CART; Stanovich et al., 2016). The ADMC is a set of six behavioral tasks measuring different aspects of decision-making (resistance to framing, recognizing social norms, overconfidence, applying decision rules, consistency in risk perception, and resistance to sunk costs) (Parker and Fischhoff, 2005; Bruine de Bruin et al., 2007)^3^. Three of the ADMC tasks can be identified as HB tasks (resistance to framing, overconfidence, and resistance to sunk costs). The full-form CART includes 20 subtests, some of them measuring HB (e.g., gambler’s fallacy, four-card selection task, and anchoring). Noteworthy, the CART and the HBI have different aims. The CART is an instrument that aims to provide an overall measure of rational thinking (the same way IQ tests measure intelligence): A given number of points is attributed to each subtest, and an overall rational thinking score (Rationality Quotient) is calculated (the full-form CART takes about 3 h to complete). Indeed, each subtest is thought to reflect a single subconstruct within the concept of rationality. Accordingly, the CART subtests are not thought to be used separately. On the other hand, the HBI follows a more basic and practical aim: Providing researchers with an open, collaborative, and evolutive inventory of HB tasks, each of which can be used separately.

### 4.3. HBI and future research

We argue that the HBI has the potential to help researchers in their investigation of several issues. The first one is the structure of rationality. Similar to other topics in psychology (e.g., intelligence, personality, executive functions, and risk preference), early studies on HB that followed an individual differences approach aimed to explore whether single or multiple factors accounted for the correlations between performance on various tasks. While some studies have suggested the existence of a single rationality factor (Stanovich and West, 1998; Bruine de Bruin et al., 2007; Erceg et al., 2022), several factor analytic studies supported multiple-factor solutions, which relate more or less to existing taxonomies of HB (e.g., Klaczynski, 2001; Weaver and Stewart, 2012; Aczel et al., 2015; Teovanović et al., 2015; Ceschi et al., 2019; Berthet, 2021; Rieger et al., 2022).

Irrespective of their results, virtually all studies that explored the structure of rationality suffered from two limitations. First, scores for some HB tasks (even multi-item ones) failed to reach satisfactory levels of reliability (e.g., Ceschi et al., 2019; Erceg et al., 2022), thereby questioning the robustness of the factorial solution. Second, the sample of HB tasks submitted to factor analysis was limited (mainly because of practical limits such as total testing duration) and not being representative of all biases listed in the literature. That limitation is important as one could reasonably expect that a higher number of tasks would result in a higher number of factors extracted. Indeed, Berthet et al. (2022) showed that there was no longer evidence of a general decision-making competence when adding four HB tasks to the six ADMC tasks while ensuring satisfactory levels of score reliability. By providing researchers with more HB tasks producing reliable scores, the HBI will further shed light on the structure of rationality. Indeed, performing factor analysis on more exhaustive samples of tasks might eventually lead to more robust empirical taxonomies of biases (Ceschi et al., 2019).

Second, the HBI will allow researchers to further address how heuristics and biases correlate with cognitive ability (Stanovich and West, 2008; Oechssler et al., 2009; Stanovich, 2012; Teovanović et al., 2015; Erceg et al., 2022; Burgoyne et al., 2023), personality traits (Soane and Chmiel, 2005; McElroy and Dowd, 2007; Weller et al., 2018), and real-life behavior (Toplak et al., 2017). Regarding the latter, Bruine de Bruin et al. (2007) reported that the ADMC components predicted significant and unique (after controlling for cognitive ability) variance on the Decision Outcome Inventory (DOI), a self-report questionnaire measuring the tendency to avoid negative real-life decision outcomes (e.g., rented a movie and returned it without having watched it at all) (refer to also Parker et al., 2015). However, Erceg et al. (2022) found no evidence that performance on HB tasks predicts various self-reported real-life decision outcomes (DOI, job and career satisfaction, peer-rated decision-making quality). In particular, personality traits (conscientiousness and emotional stability) were the most predictive of DOI scores (Berthet et al., 2022, found similar—unpublished—results). It is worth noting, however, that these studies included relatively few HB so how they relate to real-life behavior remains an issue to be further addressed.

## 5. Conclusion

As highlighted by Gertner et al. (2016, p. 3), “the study of bias within an individual difference framework is still largely in its infancy.” The present article aims to introduce the HBI, an exhaustive inventory of behavioral tasks that allow for a reliable measurement of individual differences in heuristics and biases. The aim of the HBI is to foster individual differences research in heuristics and biases by improving the visibility and accessibility of the relevant measures. As a collaborative and evolutive repository of all available measures, the success of the HBI project depends on the scientific community. Indeed, we invite researchers to support the HBI by reporting any use of the tasks (published or unpublished) and submit their own—new or alternative—measures of heuristics and biases. This open and collaborative approach will allow us to share results and continually expand the inventory.

Large-scale studies will allow to establish norm data from the general population and specific groups (e.g., documenting effects of gender and age) for each bias. Thus, our hope is that the HBI can help the research on individual differences in heuristics and biases to progress from infancy to adulthood.

## Data availability statement

The original contributions presented in the study are included in the article/supplementary material, further inquiries can be directed to the corresponding author.

## Author contributions

VB: conceptualization, methodology, and writing. VG: conceptualization and writing. All authors contributed to the article and approved the submitted version.

## Conflict of interest

The authors declare that the research was conducted in the absence of any commercial or financial relationships that could be construed as a potential conflict of interest.

## Publisher’s note

All claims expressed in this article are solely those of the authors and do not necessarily represent those of their affiliated organizations, or those of the publisher, the editors and the reviewers. Any product that may be evaluated in this article, or claim that may be made by its manufacturer, is not guaranteed or endorsed by the publisher.

## Footnotes

^1^https://sites.google.com/view/hbiproject/

^2^The original ADMC included a seventh task (path independence) which has been removed due to low reliability and validity.

^3^Studies included in the systematic review are referenced in the online material only: https://osf.io/5xg92/.
