# Supplementary material for: The heuristics-and-biases inventory: An open-source tool to explore individual differences in rationality
Source: Front Psychol. 2023 Apr 3;14:1145246. doi: 10.3389/fpsyg.2023.1145246 (PMC10106569; doi:10.3389/fpsyg.2023.1145246)
Supplement: Supplementary file 1 [file Table_1.docx]

Supplementary Material

Table 1: Full version

*Inventory and reliability of tasks measuring individual differences in heuristics and biases. Please visit the HBI website to check for updates*

| Task | Source | N items | Reliability | Task | Scoring |
| --- | --- | --- | --- | --- | --- |
| **Anchoring heuristic**  Tendency for people to adjust their (numerical) judgments towards the first piece of information. | Teovanović et al. (2015)  Replication:  Berthet (2021) | 24  12 | .77  .68 | Participants are first required to make numerical estimates (E1) relative to general knowledge. In a second phase, they are presented with the same set of items and perform a comparative task and a final estimation task. In the former, participants indicate whether the number to estimate is higher or lower than a given value (anchor, A). Anchor values are set automatically by multiplying anchor-free estimates (E1) with predetermined values (ranging from 0.2 to 1.8). Then, participants provide their final estimate (E2). | In each item, the anchoring bias is calculated as (E1 – E2) / (A – E1). Anchoring values lower than 0 (lack of anchoring) or higher than 1 (total anchoring) are removed from the analysis. The total score is the average of the scores.  Normative value: 0  Mean (SD): 0.44 (0.18) |
|  | Stanovich et al. (2016) | 8 | .48 | In each item, participants are first asked to estimate whether a quantity (e.g., the distance from San Francisco to Hawaii) is more or less than a given value (anchor) and then provide their estimate. There are four low anchors and four high anchors in the eight items. | In low anchor items, score of 1 point if estimate is above a given value and 0 point otherwise; and vice versa for high anchor items (higher scores reflect avoidance of anchoring). The total score is the sum of the scores.  Normative value: 8  Mean (SD): 3.59 (1.79) |
|  | Ceschi et al. (2019) | 5 pairs | Not reported | Participants are first asked to estimate whether a quantity is more or less than a given value (anchor) and then provide their estimate. Later on, participants are presented with the same items but with different anchor values. There are five low anchors and five high anchors. | The anchoring score is the sum of the differences between the two versions of the items.  Normative value: 10  Mean (SD): 108.34 (47.63) |
|  | Berthet, Autissier, & de Gardelle (2022) | 8 pairs | .67 (test)  .75 (retest)  Test–retest: .63 | Participants are first asked to estimate whether a quantity (e.g., the number of gold medals won by Japan at the 2012 Summer Olympics) is more or less than a given value (anchor) and then to provide their estimate. Eight pairs of items are used, where items in each pair involved similar quantities (e.g., the number of gold medals won by Japan/Australia at the 2012 Summer Olympics [actual number: 35]) but different anchors, with either a low or a high anchor corresponding to 10% or 190% of the actual number. The 16 items are presented in a random order. | In each pair, the anchoring bias score is calculated as follows: (estimate [high anchor] – estimate [low anchor]) / (high anchor – low anchor). The total score is the average of the scores.  Normative value: 0  Mean (SD): 0.47 (0.22) |
|  | Other studies | Aczel et al. (2015, 2015b); Cooper et al. (2021); Juanchich et al. (2018); Stanovich & West (2008); Teovanović (2019); Tommasi et al. (2021); Welsh et al. (2014); West et al. (2012); Yoon et al. (2021) | | | |
| **Attribution bias (including self-attribution bias)**  Tendency for people to refer to internal rather than external factors when explaining a person’s behavior. | Schneider et al. (2021) | 1 | Not applicable | The task is adapted from the paradigm of Sedikides et al. (1998) in which participants engage in two achievement tests. In each test, participants are presented with twenty anagrams and asked to solve as many as possible within 2 minutes. After each test, they receive bogus feedback about their performance: a positive feedback on one test (“You have performed better than 93% of the normative sample”) and a negative feedback on the other test (“You have performed worse than 69% of the normative sample”), regardless of their actual performance. After each test, participants indicate their agreement with four statements that provide explanations for their test results on a scale ranging from 1 (not at all) to 7 (very much). Two of the statements relate to internal factors (skills and effort) while two relate to external factors (the selection of anagrams and bad luck). | Compute (a) a score for success attributions by subtracting external attribution ratings of success from internal attribution ratings of success, (b) the equivalent score for failure attributions. The attribution bias (self-serving bias) is calculated as a – b.  Normative value: 0  Mean (SD) internal attribution ratings: 5.72 (0.99)  Mean (SD) external attribution ratings: 4.57 (1.06) |
|  | Other studies | Rieger et al. (2022); Schneider et al. (2021); van Elk (2017) | | | |
| **Availability heuristic**  Tendency for people to judge events’ likelihood or frequency based on ease of recall. | Berthet, Autissier, & de Gardelle (2022) | 4 pairs | .67 (test)  .81 (retest)  Test–retest: .48 | The task is adapted from Tversky and Kahneman (1973, Study 8). On each trial, a list of 20 items is displayed on the screen for 20s. Each list is made of items from two categories (e.g., brands of cars and drinks). Before each list, participants are informed about the forthcoming two categories and told that they will be asked to recall the number of items of one category after the presentation of the list. Pairs of lists are used such that (a) each list includes 10 items of each category (the correct answer was always 10); (b) in one list, items of one category are famous (e.g., Ferrari, Jeep, Toyota) while items of the other category are not (e.g., La Casera, Tanqueray, Glenlivet), and vice versa for the other list. Four pairs of lists are used; in each pair, the category of items to estimate is the same for the two lists. The four lists in which the items to estimate are famous are presented first, following by the four lists in which the items to estimate are not famous. | In each pair, the availability bias score is calculated as the difference between the estimate for the famous list and the estimate for the less famous list. The total score is the average of the scores.  Normative value: 0  Mean (SD): 3.33 (3.79) |
|  | Erceg et al. (2022) | 4 | .77 | The task is adapted from Lichtenstein et al. (1978). Participants are asked to indicate which of two lethal events is more common (e.g., suicide vs. diabetes). | Correct responses are scored 1 and incorrect responses 0. The total availability score is the average of the scores.  Normative value: 1  Mean (SD): 0.68 (0.30) |
|  | Ceschi et al. (2019) | 3 | Not reported | Participants rate the chance of death by two causes. | The availability bias score is the sum of the scores of the more available options.  Normative value: unknown  Mean (SD): 131.20 (49.53) |
|  | Other studies | Mušura Gabor & Gamulin (2016); Rieger et al. (2022) | | | |
| **Base-rate neglect (statistical)**  Tendency for people to ignore base rates in favor of individuating information. | Burič & Šrol (2020) | 8 | .77, .78 (calculated on conflict items) | The task is adapted from De Neys and Glumicic (2008). On each item, participants are presented with two kinds of information: base-rates (e.g., “1,000 people participated in a study, including 4 men and 996 women”) and information concerning a specific case (e.g., “Dominique is a randomly chosen participant of this study. Dominique is 23 years old and is finishing a degree in engineering. On Friday nights, Dominique likes to go out cruising with friends while listening to loud music and drinking beer”). Participants are required to decide to which one of the groups the imaginary person more likely belongs to. The task includes four no-conflict (congruent) items in which the description of the specific case fits common stereotypes of the smaller population group, and four conflict (incongruent) items in which the base-rates favour the group contrary to the stereotypical characteristic. | The score is the number of correct responses on incongruent items.  Normative value: 100%  Mean (SD): 41% (38.16) |
|  | Šrol & De Neys (2021) | 8 | .82 | Same as Burič & Šrol (2020). | |
|  | Burgoyne et al. (2021) | 11 | .46 | Same as Burič & Šrol (2020) (6 incongruent and 5 congruent items). | |
|  | Šrol (2022) | 4 | .70 | Same as Burič & Šrol (2020). | |
|  | Erceg et al. (2022) | 4 | .93, .95 | Same as Burič & Šrol (2020). | Correct responses are scored as 1 and the total score is the average of the scores.  Normative value: 1  Mean (SD): 0.58 (0.39) |
|  | Berthet (2021) | 4 | .70 | Same as Burič & Šrol (2020) but participants are asked to estimate a probability related to the specific case (“What is the probability that Dominique is a man?”). The four items used are incongruent. | The bias score is calculated as the proportion of responses that differed from the base rate information in the direction implied by the specific case (e.g., higher than 0.4% in the example).  Normative value: 0  Mean (SD): 0.53 (0.35) |
|  | Other studies | Aczel et al. (2015, 2015b); Alós-Ferrer & Hügelschäfer (2016); Alós-Ferrer et al. (2016b); Bačová & Šrol (2021); Bruckmaier et al. (2021); Cesarini et al. (2012); Ceschi et al. (2019); De Neys & Feremans (2013); Frey et al. (2018); Kaczynski (2014); Stanovich & West (1998, 1999, 2008); Thompson & Markovits (2021); Toplak & Flora (2020); Toplak et al. (2014, 2017); Vartanian et al. (2021); Weaver & Stewart (2012); Welsh & Navarro (2012); West et al. (2008, 2012) | | | |
| **Base-rate neglect (causal)**  Tendency for people to ignore causally-relevant base rates in favor of individuating information. | Teovanović et al. (2015) | 10 | .71 | Participants are provided with two conflicting pieces of information: one is statistical and favors one decision (e.g., “The probability of winning a lottery ticket is 10%”) while another is based on personal, case-based experience and favors another decision (e.g., “John, who was wearing his lucky shirt and who had found a four-leaf clover that day, decided to buy one lottery ticket”). Participants are required to estimate the probability of the criterion behavior (e.g., “What is the probability that John bought a winning ticket?”) on an 11-point percentage scale. | The total score is the proportion of responses that differed from the base rate information in the direction implied by the specific information (e.g., higher that 10% in the example).  Normative value: 0  Mean (SD): 0.78 (0.22) |
|  | Stanovich et al. (2016) | 3 | Not reported | Same as Teovanović et al. (2015) but participants choose between four possible answers: two indicating reliance on the base rate and two indicating reliance on the personal information. | Score of 1 for each of the two answers indicating reliance on the base rate, score of 0 otherwise.  Normative value: 1  Mean (SD): Not reported |
|  | Erceg et al. (2022) | 3 | .55, .48 | Same as Stanovich et al. (2016). | A continuous score (from 1 to 4) is used instead of a dichotomous one. The total score is calculated as the average of the scores.  Normative value: 4  Mean (SD): 2.96 (0.51) |
|  | Other studies | Tomljenovic et al. (2022); Toplak et al. (2011, 2017); West et al. (2008) | | | |
| **Belief bias in syllogistic reasoning**  Tendency for people to evaluate deductive arguments based on the believability of the conclusion rather than its logical validity. | Teovanović et al. (2015)  Replication:  Berthet (2021) | 8  4 | .76  -.15 | Participants are instructed to evaluate syllogisms by indicating whether the conclusion necessarily follows from the premises or not, assuming that all premises are true. The rationale is to assess the effect of the believability of the conclusion (believable vs. unbelievable) for a given level of validity of the argument (valid vs. invalid). Eight pairs of syllogisms are used, each pair involving a consistent item and an inconsistent one. On eight inconsistent (conflict) items, the logical validity of the argument is incongruent with the believability of the conclusion (two of them are valid but unbelievable, and two are invalid but believable). On eight consistent (no-conflict) items, the logical validity of the argument is congruent with the believability of the conclusion (two of them are both valid and believable, and two are both invalid and unbelievable). | The total score is the number of biased responses. A response is coded as biased if the participant provided an incorrect answer to an inconsistent item and a correct answer to the corresponding consistent item.  Normative value: 0  Mean (SD): 4.05 (2.34) |
|  | Stanovich et al. (2016) | 16 | .65 | Same as Teovanović et al. (2015) (eight consistent items and eight inconsistent items). | Raw scores range from 0 to 16 and are translated into CART points (from 0 to 8).  Normative value: 16  Mean (SD): 10.92 (2.77) |
|  | Erceg et al. (2022) | 8 | .79, .82 | Same as Teovanović et al. (2015) but only inconsistent items are used (in four of them the conclusion is valid but unbelievable; in the four others, the conclusion is invalid but believable). | The total score is the proportion of correct responses.  Normative value: 1  Mean (SD): 0.74 (0.21) |
|  | Burič & Šrol (2020) | 8 | .67, .78 (calculated on conflict items) | Same as Teovanović et al. (2015) (four consistent items and four inconsistent items). Items are based on the materials in De Neys et al. (2010). | |
|  | Šrol & De Neys (2021) | 8 | .80 (calculated on conflict items) | Same as Burič & Šrol (2020). | |
|  | Other studies | Bačová & Šrol (2021); Čavojová et al. (2020); De Neys & Van Gelder (2009); Reverberi et al. (2009); Schubert et al. (2021); Šrol (2022); Stanovich & West (1998, 2008); Stupple et al. (2011); Szászi et al. (2017); Thompson & Markovits (2021); Toplak & Flora (2020); Toplak et al. (2014, 2014b); Toplak et al. (2017); Trippas et al. (2015); Trippas et al. (2013, 2014, 2015); Tse et al. (2014); Vroling & de Jong (2013) | | | |
| **Better-than-average effect**  Tendency for people to perceive their abilities, attributes, and personality traits as superior compared with their average peer. | Rieger et al. (2022) | 3 | .60 | In each of the three items, participants indicate whether they think that their financial performance is better than others. | Each option is associated with a given number of points and the total score is the sum of the responses.  Normative value: 0  Mean (SD): 0.15 (1.48) |
|  | Other studies | Ceschi et al. (2019) | | | |
| **Bias blind spot**  Tendency for people to see themselves as less biased than other people. | Scopelliti et al. (2015) | 14 | .86 | Each item describes a bias and participants rate the extent to which they exhibit that bias and the extent to which the average American exhibits that bias on 7-point scales with endpoints, not at all (1) and very much (7). | Bias blind spot scores are calculated by subtracting the perceived self-susceptibility to each bias from the perceived susceptibility of the average American to that bias, for each bias, and then by averaging those relative differences.  Normative value: 0  Mean (SD): 1.48 (0.95) |
|  | Other studies | Toplak et al. (2014b); West et al. (2012) | | | |
| **Confirmation bias (four-card selection task)**  Tendency for people to confirm rather than infirm the hypothesis (logical rule) at hand. | Stanovich et al. (2016) | 4 | Not reported | Participants are presented with a conditional statement of “if P then Q” type and instructed to indicate which of four cards (P, not-P, Q, and not-Q) they would turn in order to test this statement (participants report whether or not they would turn over each card). The task is repeated four times, with conditional statements from slightly different contexts (two deontic and two nondeontic items). | The score is the logic index of Pollard and Evans (1987): number of correct cards turned – number of incorrect turns.  Logic index > 0 scored as 1  Logic index ≤ 0 scored as 0  Normative value: 4  Mean (SD): Not reported |
|  | Burgoyne et al. (2021) | 10 | .66 | Same as Stanovich et al. (2016) but participants are asked to select the two cards that needed to be turned over to determine whether the rule is true or false (7 items involve an abstract rule while 3 are deontic). | The total score is the number of problems answered correctly (choosing the two correct cards).  Normative value: 10  Mean (SD): 0.74 (1.29) |
|  | Erceg et al. (2022) | 4 | .86, .80 | Same as Burgoyne et al. (2021) (two deontic and two nondeontic items). | |
|  | Berthet, Teovanović, & de Gardelle (2022) | 4 | .84 | Same as Stanovich et al. (2016) but only nondeontic items are used. | In each item, the confirmation bias score is the difference between the response (Yes = 1 vs. No = 0) to the confirming card (Q) and the response to the disconfirming card (not-Q). The total score is calculated as the average of the scores.  Normative value: 0  Mean (SD): 0.42 (0.55) |
|  | Other studies | Bruckmaier et al. (2021); De Neys (2006); Stanovich & West (1998); Toplak et al. (2014b); Weaver & Stewart (2012); West et al. (2008); Witteman et al. (2009) | | | |
| **Confirmation bias (2-4-6 task)**  Tendency for people to confirm rather than infirm the hypothesis (numerical rule) at hand. | Berthet, Teovanović, & de Gardelle (2022) | 3 | .75 | Participants are provided with the following instruction: “You start a game in which your opponent has set a rule generating sequences of three numbers. Your goal is to find out this rule. For that purpose, you put forward sequences of numbers and your opponent tells you whether or not each sequence complies with the rule”. Participants are presented with a valid sequence and with the hypothesis at hand (e.g., “Your opponent starts by telling you that the sequence 2-4-6 complies with the rule. You think that ‘add 2’ is the rule”). They are also presented with two candidate sequences, one confirming (e.g., “8-10-12”) and one disconfirming (e.g., “3-6-9”). and they are asked to report whether or not they would put forward each one (Yes = 1 vs. No = 0). | In each item, the confirmation bias score is the difference between the response to the confirming sequence and the response to the disconfirming sequence. The total score is calculated as the average of the scores.  Normative value: 0  Mean (SD): 0.57 (0.55) |
| **Confirmation bias (interviewee’s personality task)**  Tendency for people to confirm rather than infirm the hypothesis (personality trait) at hand. | Berthet (2021)  Berthet, Autissier, & de Gardelle (2022)  Berthet, Teovanović, & de Gardelle (2022) | 4  4  4 | .68  .83 (test)  .88 (retest)  Test–retest: .75  .64 | The task is adapted from the employment interview task of Snyder and Swann (1978). Participants are provided with a hypothesis regarding an interviewee’s personality (e.g., the candidate is extroverted) and then select among a set of 20 questions eight ones to ask to the interviewee to test the hypothesis. The set of questions includes eight questions based on the assumption that the candidate has the personality attribute (e.g., What events make you feel popular with people?), eight questions based on the opposite assumption (e.g., What things do you dislike about loud parties?), and four neutral questions (e.g., What are some of your favorite books?). Four items are used, each one involving a particular personality trait (agreeableness, conscientiousness, emotional stability, extroversion).  In Berthet, Teovanović, and de Gardelle (2022), participants were asked to select four questions among a set of 10. | In each item, the confirmation bias score is the number of confirming questions selected by the participant. The total score is calculated as the average of the scores.  Normative value: 0  Mean (SD): 4.40 (1.37) (Berthet, Autissier, & de Gardelle, 2022) |
| **Confirmation bias (financial decision-making)**  Tendency for people to disregard the counterevidence regarding their financial investments. | Rieger et al. (2022) | 5 | .66 | The items were adapted from Pompian (2011). Participants are presented with a scenario (e.g., “Suppose you have invested in a security after some careful research. Now, you see a press release that states that the company you’ve invested in may have a problem with its main product line. The second paragraph, however, describes a completely new product that the company might debut later this year”) and they are asked to choose between different options, one reflecting the tendency to neglect the counterevidence (e.g., “I will typically take notice of the new product announcement and research that item further”). | Each option is associated with a given number of points and the total score is the sum of the responses.  Normative value: 1  Mean (SD): 3.02 (1.18) |
| **Conjunction fallacy**  Tendency for people to judge that a conjunction of two possible events is more likely than one or both of the conjuncts. | Stanovich et al. (2016) | 1 | Not applicable | Participants estimate the probability of single events (e.g., “What is the probability that you will have root canal surgery on one tooth in the next five years?) and conjunctions of two events (e.g., What is the probability that you will have root canal surgery on one tooth and another tooth extracted in the next five years?). | Root canal in five years (b) minus root canal surgery  on one tooth and another tooth extracted in five years (c) > 0 scored as 1.  Normative value: 1  Mean (SD): Not reported |
|  | Burgoyne et al. (2021) | 7 | .69 | Participants are given a scenario (e.g., the Linda problem from Tversky and Kahneman, 1983) and decide which of two statements is more likely. One of the statements (“Linda is a bank teller and is active in the feminist movement”) is a conjunction that includes the other statement (“Linda is a bank teller”). | The total score is the number of problems answered correctly.  Normative value: 7  Mean (SD): 2.33 (1.93) |
|  | Frey et al. (2018) | 2 | Not reported | One no-conflict item is used in which the single event option contains the feature which is also representative of the described individual:  Jon is 32. He is intelligent and punctual but unimaginative and somewhat lifeless. In school, he was strong in mathematics but weak in languages and art. Which one of the following statements is most likely?  Jon is an accountant  Jon is an accountant and plays in a rock band  One conflict item (such as the Linda problem) is used in which the representative feature is part of the conjunctive statement:  Jon is 32. He is intelligent and punctual but unimaginative and somewhat lifeless. In school, he was strong in mathematics but weak in languages and art. Which one of the following statements is most likely?  Jon plays in a rock band  Jon plays in a rock band and is an accountant | The score is the number of normative responses (non-conjunctive statements).  Normative value: 2  Mean: 72.8% of the whole sample gave an incorrect answer on the conflict item. |
|  | Šrol & De Neys (2021) | 8 | .78 (calculated on conflict items) | Same as Frey et al. (2018) (four no-conflict items and four conflict items). | |
|  | Šrol (2022) | 4 | .63 | Same as Šrol & De Neys (2021). |  |
|  | Other studies | Alós-Ferrer & Hügelschäfer (2016); Alós-Ferrer et al. (2016b); Bačová & Šrol (2021); Bakhti (2018); Bruckmaier et al. (2021); Cesarini et al. (2012); Ceschi et al. (2019); De Neys (2006); Juanchich et al. (2018); Klaczynski (2001); Stanovich & West (2008); Thompson & Markovits (2021); Tommasi et al. (2021); Toplak et al. (2011; 2017); Weaver & Stewart (2012); West et al. (2008; 2012); Yearsley & Trueblood (2018) | | | |
| **Conservatism**  Tendency for people to place too much weight on prior experience rather than updating beliefs with new information. | Rieger et al. (2022) | 1 | Not applicable | The item was adapted from Pompian (2011):  When you recently heard news that has potentially negative implications for the price of an investment you own, what is your natural reaction to this information?  a. I tend ignore the information. Because I have already made the investment, I’ve already determined that the company will be successful. (3) b. I will re-evaluate my reasons for buying the stock, but I will probably stick with it because I usually stick with my original determination that a company will be successful. (2)  c. I will reevaluate my reasoning for buying the stock and will decide, based on an objective consideration of all facts, what to do next. (1) | Each option is associated with a given number of points (higher number of points indicating a more pronounced bias).  Normative value: 1  Mean (SD): 3.81 (0.86) |
|  | Other studies | Mušura Gabor & Gamulin (2016) | | | |
| **Covariation detection**  Tendency for people to ignore essential comparative (control group) information. | Stanovich et al. (2016) | 25 | Not reported | Participants are presented with a 2 × 2 contingency table and are asked to judge the nature and extent of the relationship between the two variables on a 21-point scale ranging from –10 (strong negative association) to +10 (strong positive association). | The score is the correlation between the response of the subject and the true association between the variables (Δp) across the 25 items:  < 0.10: 0  ≥ 0.10 and < 0.45: 1  ≥ 0.45 and < 0.75: 2  ≥ 0.75: 3  Normative value: 1  Mean (SD): Not reported |
|  | Other studies | Toplak et al. (2007) | | | |
| **Debt account aversion**  Tendency for consumers saddled with multiple debts to be motivated to reduce their total number of outstanding loans, rather than their total debt across loans. | Ayal et al. (2011) | 1 | Not applicable | Participants are asked to imagine that they have four different credit accounts with different balances, each of which has a different annual percentage rate. They are told that they have just received a $3,000 government stimulus rebate and that they have decided to use the entire rebate to pay off debt. They must indicate how much they would allocate to each account. | Participants who considered the interest rate of each debt (a normatively irrelevant attribute) are coded as biased.  Mean: 40% of the participants gave a biased response. |
| **Denominator neglect (or ratio bias)**  Tendency for people to pay too much attention to numerators and inadequate attention to denominators. This results in a tendency to judge a low probability event as more likely when presented as a large-numbered ratio (e.g., 10/100) than as a smaller-numbered but equivalent ratio (e.g., 1/10). | Toplak et al. (2011) | 1 | Not applicable | The task is a marble game that is modeled on a task introduced by Kirkpatrick and Epstein (1992): “Assume that you are presented with two trays of black and white marbles: a large tray that contains 100 marbles and a small tray that contains 10 marbles. The marbles are spread in a single layer on each tray. You must draw out one marble (without peeking, of course) from either tray. If you draw a black marble, you win $2. Consider a condition in which the small tray contains 1 black marble and 9 white marbles, and the large tray contains 8 black marbles and 92 white marbles. [A drawing of two trays with their corresponding numbers of marbles arranged neatly in 10-marble rows appeared above the previous sentence.] From which tray would you prefer to select a marble in a real situation?” | Participants who chose the large tray are coded as biased.  Mean: 38.2% of the participants gave a biased response. |
|  | Stanovich et al. (2016) | 15 (12 scored items and  3 unscored filler items) | .88 | Same as Toplak et al. (2011) except that participants respond on 6-point Likert scale (from “Strongly prefer the small tray” to “Strongly prefer the large tray”). | |
|  | Other studies | Alonso & Fernandez-Berrocal (2003); Alós-Ferrer et al. (2016b); Ayal et al. (2011); Bačová & Šrol (2021); Čavojová et al. (2020); Kaczynski (2001b, 2014); Patel et al. (2018); Šrol (2022); Stanovich & West (2008); Stone et al. (2018); Szaszi et al. (2018); Thompson & Markovits (2021); Toplak et al. (2014, 2014b, 2017); West et al. (2008); Witteman et al. (2009) | | | |
| **Framing (risk and attribute)**  Tendency for people to be affected by how information is structured. | Bruine de Bruin et al. (2007) | 7 pairs (risk framing) and 7 pairs (attribute framing) | .62  Test–retest: .58 | The task is based on Frisch (1993), which is the first within-subject design study of the framing effect. In the risk framing task, decision problems are presented to the participants who choose between a sure-thing option (A) and a risky-choice option (B). Participants respond on a 6-point scale ranging from 1 (“I would definitely choose option A”) to 6 (“I would definitely choose option B”). Each decision problem has two versions, a gain version and a loss version. The two versions are identical, only the framing differs. In the attribute framing task, participants rate positively and negatively described versions of seven normatively equivalent events (e.g., judging the quality of ground beef labeled 80% lean or 20% fat). The gain (positive) and loss (negative) items appeared in separate blocks, with different item orders in each block. | The framing bias score is calculated as the mean absolute difference between ratings for the loss (negative) and the gain (positive) versions of each item.  Normative value: 0  Mean (SD): 3.72 (0.61) |
|  | Parker & Fischhoff (2005) | 5 pairs | .30 | Same as Bruine de Bruin et al. (2007) but a dichotomous choice is used. | Number of consistent choice pairs.  Normative value: 5  Mean (SD): 3.68 (1.09) |
|  | Stanovich et al. (2016) | 7 pairs (risk framing) and 4 pairs (attribute framing) | .66 | Same as Bruine de Bruin et al. (2007). | |
|  | Berthet (2021)  Berthet, Autissier, & de Gardelle (2022) | 8 pairs (risk framing) | .74  .76 (test)  .85 (retest)  Test–retest: .45 | Same as Bruine de Bruin et al. (2007) but the wording of the items is slightly changed between the loss and gain versions. | Consistent with prospect theory (Kahneman & Tversky, 1979), the framing bias score is calculated as the difference (rather than the absolute difference) between the mean ratings of the loss frames and the mean ratings of the gain frames.  Normative value: 0  Mean (SD): 1.00 (1.10) (Berthet, Autissier, & de Gardelle, 2022) |
|  | Erceg et al. (2022) | 4 pairs (risk framing) and 4 pairs (attribute framing) | Attribute framing: .35, .17  Risk framing: .24 | Same as Bruine de Bruin et al. (2007). | |
|  | Other studies | Attribute framing: Aczel et al. (2018); Murch & Krawczyk (2014); Saad & Gill (2014); Toplak & Flora (2020); Toplak et al. (2014); Xu et al. (2020)  Risk framing: Aczel et al. (2015, 2015b; 2018); Björklund & Bäckström (2008); Ceschi et al. (2019); Curseu (2006); Curseu & Schruijer (2012); Fang et al. (2021); Frisch (1993); Hinson et al. (2019); Rachev & Petkova (2019); Shiloh et al. (2002); Stanovich & West (2008); Toplak et al. (2011; 2014b); Weaver & Stewart (2012); West et al. (2008; 2012) | | | |
| **Fungibility of money**  Tendency for people to ignore the fact that all money is the same. | Cesarini et al. (2012) | 1 | Not applicable | The task uses slightly modified versions of the two theater ticket questions in Tversky and Kahneman (1981). Participants are asked to make a hypothetical binary choice between going to the theater or not when they had already decided to go but either lost the ticket (first question) or the money (second question) with which they had intended to buy the ticket. | Participants who gave  answers that are incompatible with fungibility (“yes” to question 1 and “no” to question 2) are coded as biased.  Mean: 5.4% of the participants were biased. |
| **Gambler’s fallacy**  Tendency for people to believe that the probability for an outcome after a series of outcomes is not the same as the probability for a single outcome. | Toplak et al. (2007) | 2 | Not reported | Participants are given a scenario (e.g., “Imagine that we are tossing a fair  coin (a coin that has a 50/50 chance of coming up heads or tails) and it has just come up heads 5 times in a row”) and decide which of three statements is correct (e.g., “It is more likely that tails will come up than heads”). | Correct responses are scored as 1.  Normative value: 2  Mean (SD): Not reported |
|  | Stanovich et al. (2016) | 5 | Not reported | Same as Toplak et al. (2007). | |
|  | Erceg et al. (2022) | 4 | .76 | Same as Toplak et al. (2007). | |
|  | Šrol (2022) | 4 | .52 | Same as Toplak et al. (2007). | |
|  | Other studies | Aczel et al. (2015); Bačová & Šrol (2021); Ceschi et al. (2019); Klaczynski (2001); Patel et al. (2018); Toplak et al. (2011, 2017); West et al. (2008) | | | |
| **Hindsight bias**  Tendency for people to make different judgments (e.g., judging the probability of an outcome) between hindsight and foresight conditions. | Teovanović et al. (2015) | 14 | .66 | In a first phase, participants perform a task in which they are asked to find the exception in a set of four words (e.g., “November,” “August,” “December,” and “January”) and then indicate the confidence in their response using a 5-point scale. Later in the test, participants receive feedback on the accuracy of each response and are asked to recall their initial confidence judgment. | The hindsight score is calculated as the proportion of hindsighted responses. A response is coded as hindsighted if the participant lowered her confidence after being informed that her response was incorrect, or raised her confidence after being informed that her response was correct.  Normative value: 0  Mean (SD): 0.33 (0.20) |
|  | Berthet (2021) | 10 | .62 | Same as Teovanović et al. (2015). | The hindsight score is calculated as (recalled confidence rating – initial confidence rating) × accuracy, with accuracy being coded as 1 (correct feedback) or -1 (incorrect feedback)  Normative value: 0  Mean (SD): 4.19 (5.77) |
|  | Other studies | Hom & Van Nuland (2019); Klaczynski (2001); Müller & Moshagen (2018); Rieger et al. (2022); Stanovich & West (1998) | | | |
| **House money effect**  Tendency for people to make decisions dependent on the prior gain or loss; includes greater tendency to gamble with recently won money. | Mušura et al. (2016) | 1 | Not applicable | The following item is used:  (1) You have just received an unexpected 3,000 kn. Choose one of the two options offered:  (a) 50% chance to earn 900 kn and a 50% chance to lose 900 kn  (b) I do not want further gains or losses  (2) You’ve just lost 3,000 kn. Choose one of the two options offered:  (a) 50% chance to earn 900 kn and a 50% chance to lose 900 kn  (b) I do not want further gains or losses | Participants who gave  response “a” to question 1 and response “b” to question 2 are coded as biased.  Mean: 17.2% of the participants were biased. |
| **Illusion of control**  Tendency for people to overestimate their ability to control events. | Rudski, J. (2004) | 1 | Not applicable | Participants are told to imagine that they have three lottery tickets:  Ticket “A” was selected with their “lucky numbers.”  Ticket “B” was found blowing down the street.  The numbers on Ticket “C” were selected by a computer.  They are then asked:  (1) which ticket they would be most likely to give away  (2) which ticket they would be most likely to keep  (3) whether or not they would exchange Ticket “A” (“lucky numbers”) for two Ticket “C’s” (computer generated).  For the first two questions, participants are given the option of responding "no preference." | Participants who reported that they would not exchange the ticket A are coded as biased.  Mean: 43% of the participants were biased. |
|  | Other studies | Rieger et al. (2022) | | | |
| **Insensitivity to sample size**  Tendency for people to neglect that a larger sample size is more likely to approximate a population value. | Toplak et al. (2011) | 2 | Not reported | The first item is the Hospital problem used by Tversky and Kahneman (1974):  There are two hospitals in a city. In the big hospital, 45 children are born every day, and in the small hospital 15 children are born every day. On average, 50% of the children born are boys, but it varies from day to day. In which hospital do you think that it is most likely that more than 60% of the children born are boys in a specific day?  The big hospital  The small hospital  The second item is the Squash problem used by Kahneman and Tversky (1982):  As you know, a game of squash can be played either to 9 or to 15 points. Holding all other rules of the game constant, if A is a better player than B, which scoring system will give A a better chance of winning?  The 9-point scoring system  The 15-point scoring system | The biased response in the Hospital problem is “The big hospital” and the 9-point  scoring system in the Squash problem.  Mean: 71.4% (84.4%) of the participants gave a biased response in the Hospital Problem (Squash problem). |
|  | Other studies | Aczel et al. (2015; 2015b); Cesarini et al. (2012); Stanovich et al. (2016); Patel et al. (2018); Toplak et al. (2017) | | | |
| **Irrational diversification**  Tendency for people to favor a portfolio based on the perceived risk rather than the actual risk of the portfolio (based on real variance  or probability). | Ayal et al. (2011) | 1 | Not applicable | The following item is used:  Imagine five stacks of lottery tickets. Each ticket has the numbers 1 to 49. A computer will randomly select five different numbers from this range. In order to win a monetary prize, you are required to guess the numbers that will come up. To take part in the bet, please select method A or method B below:  (a) Method A: Pick five tickets and mark five numbers on each of them. If you mark at least one of your tickets with the five numbers that are  randomly selected in the lottery, you win.  (b) Method B: Pick one lottery ticket and mark six numbers from the entire range. If you mark the five numbers that are randomly selected in the lottery, you win. | Participants who choose the portfolio with higher  perceived diversity but lower expected value (Method A) are coded as biased.  Mean: 47% of the participants were biased. |
| **Loss aversion**  Tendency to prefer avoiding losses to acquiring equivalent gains. | Gächter et al. (2022) | 1 | Not applicable | Loss aversion is measured at the individual level in riskless and risky choices. The riskless task is an endowment effect experiment in which WTA and WTP are elicited. In the risky choice task, participants are presented with six low-stake lotteries with a 50-50 chance of a fixed gain (€6) and losses that varied from €2 to €7 and they indicate for each one whether they would play the lottery or not. | In the riskless task, loss aversion is the difference between WTA and WTP. In the risky task, participants who monotonically rejected the lotteries with non-negative payoffs (those with losses from €2 to €5) are coded as loss averse.  Mean: 82% (71%) of the participants displayed loss aversion in riskless (risky) choice. |
|  | Other studies | Cesarini et al. (2012); Hermann (2017); Mušura Gabor & Gamulin (2016) | | | |
| **Mental accounting**  Tendency to assign different mental values to the same sum of money. | Rieger et al. (2022) | 1 | Not applicable | The item was adapted from Pompian (2011):  Part A: Suppose you are at a big store, where you intend to purchase an oven. The model you’ve selected is priced at is 2000 TWD, and you are about to pay. However, at the last minute, you notice an advertisement flyer featuring the same oven, at a price of 1700 TWD. The discount offer is only valid for today. You’ll need to drive 10 min to buy it in a competing store. Would you like to take the bus to the other store to take advantage of the lower price?  (a) Yes  (b) No  Part B: Now suppose you are in the same store, this time to buy a refrigerator. The refrigerator you want costs 30,000 TWD, and you are willing to pay. While you are waiting, you strike up a conversation with another store patron, who reveals that she has seen the same refrigerator available for 29,700 TWD at a competing local store about 10 min drive away. Will you drive to the other store to obtain the lower price?  (a) Yes  (b) No | Participants who selected “a” in part A and “b” in part B are coded as biased.  Mean: 79% of the participants were biased. |
|  | Other studies | Tommasi et al. (2021) | | | |
| **Money illusion**  Tendency for people to think of money in nominal, rather than real, terms. | Tommasi et al. (2021) | 1 | Not applicable | The item was taken from Shafir et al. (1997):  Suppose Adam, Ben, and Carl each received an inheritance of $200,000, and each used it immediately to purchase a house. Suppose that each of them sold the house a year after buying it. Economic conditions, however, were different in each case:  * When Adam owned the house, there was a 25% deflation—the prices of all goods and services decreased by approximately 25%. A year after Adam bought the house, he sold it for $154,000 (23% less than he paid).  * When Ben owned the house, there was no inflation or deflation—prices had not changed significantly during that year. He sold the house for $198,000 (1% less than he paid for it).  * When Carl owned the house, there was a 25% inflation—all prices increased by approximately 25%. A year after he bought the house, Carl sold it for $246,000 (23% more than he paid).  Please rank Adam, Ben, and Carl in terms of the success of their house-transactions. Assign 1 to the person who made the best deal, and 3 to the person who made the worst deal. | Participants who chose Carl as the person who made the best deal (the only one to make a nominal gain but  a real loss) are coded as biased.  Mean: 58.18% of the participants were biased. |
| **Myside bias**  Tendency for people to evaluate evidence, generate evidence, and test hypotheses in a manner biased toward their own prior opinions and attitudes. | Stanovich & West (2008b) | 1 | Not applicable | Participants first respond to a questionnaire item, embedded in a series of other items, that probes the participant’s position on the issue of abortion (“I believe that abortion should be legal in this country”). Then, each participant grades four paragraphs: two two-sided paragraphs and two one-sided paragraphs (one on each side of the abortion issue). | Each participant receives four scores: the rating (numerically transformed grade) they gave their 4anti/0pro paragraph, the rating they gave their 0anti/4pro paragraph, the rating they gave their 2anti/2pro paragraph, and the rating they gave their 2pro/2anti paragraph. Each participant’s myside bias is indexed by a difference score whose calculation depends on their prior opinion (e.g., for the anti-abortion participants, the myside bias score is derived by subtracting their 0anti/4pro score from their 4anti/0pro score).  Normative value: 0  Mean (SD): 1.40 (3.98) |
|  | Other studies | McCrudden & Barnes (2016); West et al. (2012); Wolfe (2012) | | | |
| **Omission bias**  Tendency for people to avoid actions that carry some risk but that would prevent a larger risk. | Stanovich & West (2008) | 1 | Not applicable | Participants read the following problem:  Imagine that there will be a deadly flu going around your area next winter. Your doctor says that you have a 10% chance (10 out of 100) of dying from this flu. However, a new flu vaccine has been developed and tested. If taken, the vaccine prevents you from catching the deadly flu. However, there is one serious risk involved with taking this vaccine. The vaccine is made from a somewhat weaker type of flu virus, and there is a 5% (5 out of 100) risk of the vaccine causing you to die from the weaker type of flu. Imagine that this vaccine is completely covered by health insurance. If you had to decide now, which would you choose?  1. I would definitely not take the vaccine. I would thus accept the 10% chance of dying from this flu. [scored as 1]  2. I would probably not take the vaccine. I would thus accept the 10% chance of dying from this flu. [scored as 2]  3. I would probably take the vaccine. I would thus accept the 5% chance of dying from the weaker flu in the vaccine. [scored as 3]  4. I would definitely take the vaccine. I would thus accept the 5% chance of dying from the weaker flu in the vaccine. [scored as 4] | Each option is associated with a given number of points (higher number of points indicating a less pronounced bias).  Mean: 25.6% (30.3%) of the participants displayed some degree of omission bias in the high-SAT (low-SAT) group. |
|  | Other studies | Tomljenovic et al. (2022) | | | |
| **Outcome bias**  Tendency for people to evaluate the quality of a decision based on its outcome. | Teovanović et al. (2015) | 10 pairs | .83 | In the first phase, participants are presented with descriptions of 10 scenarios describing a decision made by an individual (e.g., “Two seconds before the end of the basketball match, with the score 79 to 77 in favor of the opponent, a player of the Serbian national basketball team decided to make a shoot that would earn three points”) and the outcome of the decision, that was either positive (e.g., “and he scored”) or negative (e.g., “and he missed”). Participants are asked to judge the quality of the decision by indicating whether the decision maker should make the same choice in similar situations on a rating scale ranging from 1 (absolutely not) to 6 (absolutely yes). A week later, a parallel form of the questionnaire is administered, with the same 10 decisions but with different outcomes: if the outcome was positive in the first test, it is negative in the second, and vice versa. | The outcome bias score is calculated as the difference between the mean ratings of decisions with positive outcomes and the mean ratings of decisions with negative outcomes.  Normative value: 0  Mean (SD): 1.55 (0.91) |
|  | Berthet (2021)  Berthet, Autissier, & de Gardelle (2022) | 16 | .85^b^  .89^b^ (test)  .91^b^ (retest)  Test–retest: .78 | Participants are presented with various scenarios (e.g., a physician who decided to go ahead with an operation) and rate the quality of the decision on a 6-point scale ranging from 1 (“It was a poor decision”) to 6 (“It was an excellent decision”). Eight items involve a positive outcome (e.g., “The operation succeeded”) and eight items involve a negative one (e.g., “The patient died”). Different scenarios are used for the two outcome conditions. | Same as Teovanović et al. (2015).  Normative value: 0  Mean (SD): 2.99 (2.88) (Berthet, Autissier, & de Gardelle, 2022) |
|  | Erceg et al. (2022) | 4 pairs | .65, .68 | Same as Teovanović et al. (2015) but each pair includes parallel, relatively equivalent scenarios (one with a positive and the other with a negative outcome). | |
|  | Other studies | Aczel et al. (2015, 2015b); Klaczynski (2001); Patel et al. (2018); Stanovich & West (1997, 1998, 2008); Tomljenovic et al. (2022); Toplak et al. (2007, 2011); West et al. (2012) | | | |
| **Overconfidence**  Tendency for people to overestimate their abilities. | Bruine de Bruin et al. (2007) | 34 | .77  Test–retest: .47 | Participants respond to a performance task (dichotomous general knowledge items) and then indicate the confidence in their response on a 6-point scale ranging from 50% (“I am just guessing”) to 6 (“I absolutely sure”). | Overconfidence is assessed through a calibration measure, defined as the difference between the mean confidence ratings and the mean accuracy (percentage of correct answers).  Normative value: 0  Mean (SD): 0.91 (0.08) |
|  | Parker & Fischhoff (2005) | 42 | .79 | Same as Bruine de Bruin et al. (2007). | |
|  | Teovanović et al. (2015) | 21 | .94 | Same as Bruine de Bruin et al. (2007) but the performance task is the Letter Counting test and participants indicate the confidence in their response on an 11-point rating scale, ranging from 0% to 100% in steps of 10%. | |
|  | Stanovich et al. (2016) | 36 | .55 | Same as Bruine de Bruin et al. (2007) but participants indicate the confidence in their response on a 6-point rating scale, ranging from 50% (“I was just guessing”) to 100% (“I am certain”) in steps of 10%. | |
|  | Berthet (2021)  Berthet, Autissier, & de Gardelle (2022) | 11 | .81^b^  .73^b^ (test)  .59^b^ (retest)  Test–retest: .54 | Same as Teovanović et al. (2015) but the performance task is the matrix reasoning task from the International Cognitive Ability Resource (Condon & Revelle, 2014). | |
|  | Hansson et al. (2008) | 40 for each task | .84 (interval production task)  .80 (probability judgment task) | In the interval production task, the participants are first requested to make their best guess about the population of a country. Thereafter, they produce an 80% intuitive confidence interval believed to include the true population figure with the stated probability. For this task, the following wording is used: “Produce the (smallest) interval within which you are 80% certain that the population of Burma falls between ____ and ____million.”  In the probability judgment task, the participants make a best guess about the population of a country and then are asked to assess the probability that a prestated interval would include the true population figure. For this task, the following wording is used (X defines the lower limit and Y the upper limit of a prestated interval): “The population of Burma lies between X and Y million. What is the probability that this statement is correct: 0%, 10%, 20%, 30%, 40%, 50%, 60%, 70%, 80%, 90%, or 100%?” | In the interval production format (OCIP), the overconfidence score is calculated as the proportion of the produced intervals that included the correct value subtracted from the prestated confidence level (i.e., 80%). In the probability judgment format (OCPJ), the proportion of the predefined intervals that included the correct value was subtracted from mean probability judgment (i.e., ranging between 0% and 100%). In both cases, zero defined perfect calibration, a positive score represented overconfidence, and a negative score represented underconfidence.  Mean (SD) for OCIP: 0.48 (0.16)  Mean (SD) for OCPJ: 0.07 (0.13) |
|  | Glaser et al. (2013) | 15 | .83 | The authors introduced a new method to measure overconfidence in interval estimates, which is based on the implied probability mass behind a stated prediction interval. Participants observe artificially generated stock price charts that have, in the long run, either a positive or negative trend. They are asked to predict the future price development of these time-series charts via confidence intervals (the task is quite complex, see the original article for further details). | Overconfidence is measured via the (induced) probability  mass covered by the stated prediction interval. |
|  | Other studies | Aczel et al. (2015b); Busenitz & Barney (1997); Cheng et al. (2021); Hansson et al. (2008b); Juanchich et al. (2018); Stanovich & West (1998) | | | |
| **Probability matching (vs. maximizing)**  Tendency for people to match choice proportions to outcome proportions in a binary prediction task. | West & Stanovich (2003) | 2 | Not reported | First item:  A die with 4 red faces and 2 green faces will be rolled 60 times. Before each roll you will be asked to predict which color (red or green) will show up once the die is rolled. Pretend that you will be given 1 dollar for each correct prediction. Assume that you want to make as much money as possible. What strategy would you use in order to make as much money as possible by making the most correct predictions?  Strategy A: Go by intuition, switching when there has been too many of one color or the other.  Strategy B: Predict the more likely color (red) on most of the rolls but occasionally, after a long run of reds, predict a green.  Strategy C: Make predictions according to the frequency of occurrence (four of six for red and two of six for green). That is, predict twice as many reds as greens.  Strategy D: Predict the more likely color (red) on all of the 60 rolls.  Strategy E: Predict more red than green, but switching back and forth depending upon “runs” of one color or the other.  Second item:  A card deck has only 10 cards. Seven of the cards have the letter “a” on the down side. Three of the cards have the letter “b” on the down side. The 10 cards are randomly shuffled. Your task is to guess the letter on the down side of each card before it is turned over. Pretend that you will win $100 for each card’s down side letter you correctly predict. Indicate your predictions for each of the 10 cards. | In the first item, score of 1 if the participant chose Strategy D (maximizing strategy), all other options are scored as 0.  In the second item, score of 1 if all responses follow the normative strategy of maximizing (e.g., predicting “a” for all cards). Any other response is non-normative and scored as 0.  Mean: 47.2% (33.5%) of the participants chose the maximizing strategy in the dice (card) problem. |
|  | Fletcher et al. (2011) | 2 | .68 | Same as West & Stanovich (2003). | |
|  | Other studies | Patel et al. (2018); Stanovich et al. (2016); Stanovich & West (2008); Toplak et al. (2007, 2011, 2017) | | | |
| **Probability neglect bias**  Tendency for people to disregard the small probability of an outcome when facing a situation that arouses strong emotions. | Shimizu et al. (2022), Suter et al. (2016) | 1 | Not applicable | Please refer to the original papers for the detailed method and scoring. | |
| **Proportion dominance**  Preference for proportionally higher gains, such that the same absolute quantity is valued more as the reference group decreases (e.g., saving 10/10 lives is preferred to saving 10/100 lives). | Bartels (2006) | 5 | Not reported | In each item, two interventions are described as treating identical problems in two different populations. The (joint evaluation) items are constructed by including both a small and a large reference group. Participants are asked to rate their relative strength of preference for one intervention over the other by circling one of seven partitioning marks along a scale bounded by “Strong Preference for Program A” and “Strong Preference for Program B”.  Example: Anthrax powder has been weaponized and released into the air above two mid-sized cities. In each city, a number of people are expected to die as a result of anthrax inhalation. There exists a powerful antibiotic that will successfully treat some victims, but there is a limited amount of this treatment. Program A would delegate the treatment to City A, and 225 of the 300 at risk of death will be saved. Program B would delegate the treatment to City B, and 230 of the 920 people at risk of death will be saved. These programs are mutually exclusive and the only two options available. | Responses at the end of the scale corresponding to a strong preference for the intervention that maximized absolute savings are coded as zeros; responses at the end of the scale corresponding to a strong preference for the intervention maximizing relative savings are coded as ones. This yields a measure of proportion dominance in joint evaluation, with responses coded by their difference (from zero to one) from the absolute savings end of the scale.  Mean: 48% of participant’s responses were greater than 0.5. |
| **Regression to the mean**  Tendency for people to neglect that extremely high or extremely low observations tend to become more moderate (i.e., closer to the mean) over time. | Toplak et al. (2007) | 1 | Not applicable | The item was taken from Lehman et al. (1988):  After the first 2 weeks of the major league baseball season, newspapers begin to print the top 10 batting averages. Typically, after 2 weeks, the leading batter often has an average of about 0.450. However, no batter in major league history has ever averaged 0.450 at the end of the season. Why do you think this is? Circle one:  a. When a batter is known to be hitting for a high average, pitchers bear down more when they pitch to him.  b. Pitchers tend to get better over the course of a season, as they get more in shape. As pitchers improve, they are more likely to strike out batters, so batters’ averages go down.  c. A player’s high average at the beginning of the season may be just luck. The longer season provides a more realistic test of a batter’s skill.  d. A batter who has such a hot streak at the beginning of the season is under a lot of stress to maintain his performance record. Such stress adversely affects his playing.  e. When a batter is known to be hitting for a high average, he stops getting good pitches to hit. Instead, pitchers ‘‘play the corners’’ of the plate because they don’t mind walking him. | Score of 1 if the participant chose the normative response (c), all other options are scored as 0.  Normative value: 1  Mean (SD): Not reported |
|  | Other studies | Aczel et al. (2015, 2015b); Ceschi et al. (2019); Stanovich et al. (2016); Toplak et al. (2011, 2017); West et al. (2008) | | | |
| **Regret aversion**  Tendency for people to make decisions in order to avoid feeling regret in the future. | Rieger et al. (2022) | 3 | .60 | The items were adapted from Pompian (2011). Participants are presented with a scenario (e.g., “Suppose you invest in company A’s stock and over the next 12 months the stock price appreciates by 10 percent. You contemplate selling stock A for normal portfolio rebalancing purpose, but then come across positive news about the company in the economic daily. It is mentioned that the stock price has a chance to increase further in the near future.”) and they are asked to choose between different options, one reflecting the tendency to make decisions in order to avoid feeling regret in the future (e.g., “I think I’ll hold off and sell later. I’d really kick myself if I sold now and stock A continued to go up”). | Each option is associated with a given number of points and the total score is the sum of the responses.  Normative value: 0  Mean (SD): 2.01 (0.97) |
| **Representativeness heuristic**  Tendency for people to assess similarity of objects and organize them based around the category prototype. Some studies have investigated biases produced by this heuristic in a grouped manner. | Yoon et al. (2021) | 26 | .90 (subscale A)  .86 (subscale B)  .88 (subscale C) | Each subscale includes items measuring the conjunction fallacy, base rate neglect, gambler’s fallacy, misperception of randomness, and insensitivity to sample size. | Statistically or logically correct answers are scored as 1, and incorrect (biased) answers are scored as 0.  Normative value: 1  Mean (SD): Not reported |
|  | Morsanyi et al. (2009) | 3 | .51 | One item measures the gambler’s fallacy, a second item measures misperception of randomness, and a third item measures the conjunction fallacy. | Statistically or logically correct answers are scored as 1, and incorrect (biased) answers are scored as 0.  See Table 1 |
|  | Other studies | Busenitz & Barney (1997); Ceschi et al. (2019); Curseu (2006); Curseu & Schruijer (2012); Mušura Gabor & Gamulin (2016); Tommasi et al. (2021) | | | |
| **Status quo bias (or default bias)**  Tendency for people to choose the default option. | Cesarini et al. (2012) | 3 | Not reported | The three items are as follows:  1. A few years ago it became possible to switch electricity provider. Have you switched electricity provider since this possibility was introduced?  Yes/No  2. A few years ago it became possible to switch telephone operator from Telia. Have you switched telephone operator since this possibility was introduced?  Yes/No  3. Have you left the Church of Sweden since the separation from the state?  Yes  No, I am still in the Church of Sweden  No, I did not belong to the Church of Sweden at the separation from the state | Each response is coded with the value 1 if the individual reported a change of provider and 0 otherwise. Similarly, individuals who reported having left the Church of Sweden are coded as 1, and individuals who reported not having left are coded as 0. Compute an index of default bias by summing the responses to the three questions (a higher number denotes less susceptibility to status quo bias).  Normative value: 0  Mean (SD): 1.095 (0.890) |
|  | Other studies | Mušura Gabor & Gamulin (2016) | | | |
| **Sunk cost fallacy**  Tendency for people to continue an endeavor once an investment in money, effort, or time has been made. | Bruine de Bruin et al. (2007)  Replication:  Berthet (2021)  Berthet, Autissier, & de Gardelle (2022) | 10  5  10 | .54  Test–retest: .61  .35  .38 | Participants are presented with hypothetical scenarios (e.g., “After a large meal at a restaurant, you order a big dessert with chocolate and ice cream. After a few bites you find you are full and you would rather not eat any more of it”) and choose between the sunk-cost option (e.g., “Most likely to eat more”) and the normatively correct option (e.g., “Most likely to stop eating”) using a 6-point scale ranging from 1 (the normatively correct option) to 6 (the sunk-cost option). | The sunk cost fallacy score is the mean rating score.  Normative value: 1  Mean (SD): 4.40 (0.77) |
|  | Parker & Fischhoff (2005) | 2 | .03 | Same as Bruine de Bruin et al. (2007). | Number of times resisted sunk cost.  Normative value: 2  Mean (SD): 0.80 (0.70) |
|  | Teovanović et al. (2015) | 8 | .76 | Same as Bruine de Bruin et al. (2007). | |
|  | Erceg et al. (2022) | 4 | .56, .39 | Same as Bruine de Bruin et al. (2007). | |
|  | Other studies | Aczel et al. (2015, 2015b); Bruine de Bruin et al. (2014); Ceschi et al. (2019); Frisch (1993); Kaczynski (2001b); Patel et al. (2018); Stanovich & West (1999); Strough et al. (2008); Toplak et al. (2011) | | | |
| **Temporal discounting**  Tendency for people to prefer the smaller immediate reward in favor of a larger delayed reward. | Toplak et al. (2014b) | 5 | Not reported | The task is adapted from Frederick (2005). For each item, participants indicate the strength of their preference for either a smaller amount of money now or a larger amount of money later (e.g., “I prefer $3400 this month or $3800 next month”). In each case the delayed larger amount corresponds to a substantial percentage increase in value, which on a simple interest basis would result in value increases of between 40% to 240% if earned annually. Participants indicated their preferences using a 6-point scale ranging from 1 (e.g., “I strongly prefer $3400 this month) to 6 (e.g., “I strongly prefer $3800 next month”). | The temporal discounting score is the sum of the responses to the five items (a higher score indicates a preference to wait for the larger amount of money).  Normative value: 30  Mean (SD): 17.82 (6.27) |
|  | Stanovich et al. (2016) | 26 | .97 | Same as Toplak et al. (2014b). | |
|  | Other studies | Basile & Toplak (2015); Cesarini et al. (2012); Ceschi et al. (2019); Da Silva et al. (2017); Juanchich et al. (2018); Mørkbak et al. (2017); Toplak & Flora (2020); Toplak et al. (2017); van den Bos et al. (2014) | | | |

*Note.* Reliability is measured by Cronbach’s alpha or Spearman-Brown corrected split-half reliability (^b^). In the “Scoring” column, we report the normative value (the expected score for unbiased participants) and the average score across participants reported in the original study. Regarding the tasks of the CART, the reliability values reported are those of the Full-Form (Stanovich et al., 2016). Regarding confirmation bias, we reported measures of the information search component, which are the most common (see Berthet, Teovanović, & de Gardelle, 2022, for measures of the weighing of evidence and memory recall components).
